# *Lycium* *barbarum* Polysaccharide (LBP): A Novel Prebiotics Candidate for *Bifidobacterium* and *Lactobacillus*

**DOI:** 10.3389/fmicb.2018.01034

**Published:** 2018-05-18

**Authors:** Fang Zhou, Xiaoying Jiang, Tao Wang, Bolin Zhang, Hongfei Zhao

**Affiliations:** ^1^Department of Food Science, College of Biological Science and Biotechnology, Beijing Forestry University, Beijing, China; ^2^Beijing Key Laboratory of Forest Food Processing and Safety, Beijing Forestry University, Beijing, China

**Keywords:** *Lycium barbarum* polysaccharides, proliferation, transcriptome, stress conditions, activity

## Abstract

*Lycium barbarum* is a boxthorn that produces the goji berries. The aim of the current study was to evaluate the proliferative effect of *L. barbarum* polysaccharides (LBP) on probiotics. LBP was extracted from goji berries and its monosaccharide composition characterized by gas chromatography (GC). The LBP extract contained arabinose, rhamnose, xylose, mannose, galactose, and glucose. LBP obviously promoted the proliferation of lactic acid bacteria (LAB) strains, especially *Bifidobacterium longum* subsp. *infantis* Bi-26 and *Lactobacillus acidophilus* NCFM. In the presence of LBP in the growth medium, the β-galactosidase (β-GAL) and lactate dehydrogenase (LDH) activities of strain Bi-26 significantly increased. The activities of β-GAL, LDH, hexokinase (HK), 6-phosphofructokinase (PFK), and pyruvate kinase (PK) of strain NCFM significantly increased under those conditions. LAB transcriptome sequencing analysis was performed to elucidate the mechanism responsible for the proliferative effect of LBP. The data revealed that LBP promoted the bacterial biosynthetic and metabolic processes, gene expression, transcription, and transmembrane transport. Pyruvate metabolism, carbon metabolism, phosphotransferase system (PTS), and glycolysis/gluconeogenesis genes were overexpressed. Furthermore, LBP improved cell vitality during freeze-drying and tolerance of the gastrointestinal environment. In summary, LBP can be used as a potential prebiotic for *Bifidobacterium* and *Lactobacillus*.

## Introduction

Lactic acid bacteria (LAB) are important probiotics. Their beneficial effect on the host health has been widely recognized (Steinkraus, 2002). Because of their metabolic activities and fermentation products, LAB exert many physiological functions, e.g., immunostimulation, improved digestion and absorption, vitamin synthesis, inhibition of the growth of potential pathogens, cholesterol reduction, and reduction of gas distension (Wallace et al., 2011; Pandey et al., 2015). Therefore, LAB are widely used in the food industry (Corbo et al., 2014). Before LAB reach and colonize the human large intestine to fulfill their physiological functions, they must retain their viability during the following three stages: (i) storage; (ii) industrial processing, such as freeze-drying; and (iii) transit through the stomach and the small intestine (Figueroa-Gonzalez et al., 2011).

Prebiotics are usually non-digestible substances that exert positive effects on probiotic proliferation and host health. They are mainly oligosaccharides, polysaccharides, protein hydrolyzates, short-chain fatty acids, and plant and herb extracts (Wang et al., 2015). Supplementation of the growth medium with prebiotics is an effective way of promoting the proliferation of LAB and improving their viability under challenging conditions.

LAB strains can use various carbohydrates, a type of prebiotic, to produce energy required for their proliferation. Further, carbohydrates contain many hydroxyl groups; hence, they can form hydrogen bonds with polar groups on proteins to ensure protein stability, which could increase cell survival. Most prebiotics are short-chain carbohydrates with a degree of polymerization of two or more, e.g., inulin, fructooligosaccharides, and galactooligosaccharides (Figueroa-Gonzalez et al., 2011). Several studies have shown that some plant polysaccharides could be used as prebiotics, e.g., ginseng polysaccharides, *Mangifera pajang* fibrous polysaccharides, mushroom polysaccharides, and coconut polysaccharides (Al-Sheraji et al., 2012; Chou et al., 2013; He et al., 2015; Mohd Nor et al., 2017).

*Lycium barbarum* is a boxthorn that produces the goji berries. Goji berry is a traditional Chinese food material that can be used as a foodstuff and medicine. Polysaccharides are the most abundant group of active ingredients of the goji berry; they are composed of several types of monosaccharides (Kulczyński and Gramza-Michałowska, 2016). Currently, *L. barbarum* polysaccharides (LBP) are only infrequently used as prebiotics. Thus, the objectives of the current study were to: (i) characterize the monosaccharide composition of LBP; (ii) evaluate the effect of LBP on LAB proliferation; (iii) explore the mechanism of the proliferation by transcriptome analysis; and (iv) evaluate the protective effect of LBP on LAB under challenging conditions.

## Materials and Methods

### Preparation of LBP

Dry fruits of *L. barbarum* were provided by Ningxia Senmiao Technology Group Co., Ltd. (Yin Chuan, China). Overall, 2 kg goji berries were used in the current study. Polysaccharides were extracted as described by Skenderidis et al. (2017) with some modification. Briefly, dry fruits were softened by soaking in distilled water (1:10, w/v) and then broken by using a juice extractor (Jiuyang, Shandong, China). The pulp was extracted with distilled water (1:20) for 3 h at 80°C, centrifuged (2000 × *g* for 10 min) to remove the fruit residue, and the supernatant vacuum-concentrated at 80°C. The concentrate was then mixed with ethanol (95%), and stored at 4°C for 12 h. Finally, the precipitates were collected and freeze-dried, for a crude LBP preparation. The polysaccharides were purified (pigment and protein removed) by passing through a macroporous resin S-8 (Jinkai, Shanghai, China), as described by Yang et al. (2012).

### Gas Chromatography (GC) Analysis of LBP

Glucose, arabinose, rhamnose, mannose, galactose, and xylose standards were provided by the National Standard Substances Center (Beijing, China). The polysaccharide content was determined by the anthrone–sulfuric acid method, using glucose as a standard (Dubois et al., 1956; Ren et al., 2013).

The monosaccharide composition of LBP was analyzed by GC. The LBP sample (5 mg) was hydrolyzed in 4 mL of trifluoroacetic acid (4 M) at 110°C for 3 h. The trifluoroacetic acid was removed by evaporation with anhydrous ethanol. The hydrolyzate was derivatized by incubation with pyridine (1.0 mL), hexamethyldisilazane (HMDS; 0.6 mL), and trimethylchlorosilane (0.3 mL), at 50°C for 1 h (Song et al., 2011; Li et al., 2014). The samples were analyzed by GC (GC-2010, Shimadzu, Japan) using a Shimadzu capillary column OV-1701 (30 m × 0.25 mm × 0.33 μm). The analytical conditions were as follows: 0–5 min at 180°C; ramp up 180°C to 230°C at 10°C/min; and hold for 20 min at 230°C. The monosaccharides were quantified using an external standard method.

### Bacterial Strains and Culture Condition

The LAB strains used in the current study were five *Bifidobacterium* strains: *B. bifidum* Bb-02, *B. animalis* subsp. *lactis* Bi-04, *B. longum* subsp. *infantis* Bi-26, *B. longum* subsp. *longum* A6, and *B. animalis* BY-02; and two *Lactobacillus* strains: *L. acidophilus* NCFM and *L. plantarum* LP39. *L. plantarum* LP39 was obtained from the China Center of Industrial Culture Collection (CICC), while the other strains were provided by DuPont Co., Ltd. (Shanghai, China). *Lactobacillus* strains were cultured on de Man, Rogosa, and Sharpe (MRS) medium, while the *Bifidobacterium* strains were cultured anaerobically in MRS medium containing 0.05% L-cysteine (Sigma-Aldrich, St. Louis, MO, United States). All strains were incubated at 37°C for 24 h. The cells were collected by centrifugation (4°C, 6000 × *g*, 5 min), washed twice with sterile saline solution, and resuspended in an equal volume of sterile saline before inoculation.

### Effect of LBP on the Proliferation of LAB Strains

The effect of LBP on the proliferation of LAB strains was evaluated in three media. These were as follows: (i) control check (CK) medium, unsupplemented MRS medium; (ii) G1 medium, MRS medium supplemented with 0.5% LBP; and (iii) G2 medium, MRS medium containing 5% LBP instead of 5% glucose. Cell viability was confirmed by plate-counting on MRS agar after 37°C for 48 h. For *Bifidobacterium* strains, L-cysteine (0.05%) was added to the medium. All experiments were carried out in triplicate.

### Effect of LBP on the Activities of Key Glucose Metabolism Enzymes

The activities of key glucose metabolism enzymes were determined as follows. For *Bifidobacterium* strains, β-galactosidase (β-GAL) and lactate dehydrogenase (LDH) activities were determined. For *Lactobacillus* strains, the activities of β-GAL, LDH, hexokinase (HK), pyruvate kinase (PK), and 6-phosphofructokinase (PFK) were determined. Proteins were extracted by using the One Step Bacterial Active Protein Extraction kit (Sangon Biotech, Shanghai, China) and quantified using BCA Protein Assay kit (Sangon Biotech). The activities of key enzymes were determined using the appropriate enzyme activity kits according to the manufacturer’s protocols (Suzhou Comin Biotechnology, Jiangsu, China). The enzyme activity was expressed as units (U) of activity per mg protein (U/mg prot).

### Transcriptome Sequencing

In order to investigate the activation mechanism resulting from the presence of LBP, cells from G1 medium were analyzed and cells cultured in CK medium were used as control. Total RNA was extracted using the Trizol reagent (Invitrogen, Carlsbad, CA, United States). The rRNA was removed from RNA samples using the mRNA-only kit (Epicentre Biotechnologies, WI, United States). RNA concentration and purity were determined using Agilent 2100 Bioanalyzer (CA, United States). The mRNA was isolated from total RNA using Sera-Mag Magnetic oligo(dT) particles, and then chemically fragmented. The sequence library was constructed according to instructions for the ScriptSeq^TM^ mRNA-Seq Library Preparation kit (Illumina-compatible). Samples were sequenced simultaneously using a single flow cell of the Illumina Hiseq2000 (CA, United States).

Quality reads were assembled into contigs, transcripts, and unigenes using the Velvet and Oases software. The reads per kilobase of exon model per million mapped reads value (RPKM) was used to normalize transcript abundances. A twofold differential was used to identify differentially expressed genes. All unigenes were used as queries in searching the Nr and SwissProt databases, and functionally annotated by GO analysis using the Blast2GO software^1^. Metabolic pathways were predicted by KEGG mapping.

### Effect of LBP on LAB Strains Exposed to Stress Conditions

To explore the protective effect of LBP on LAB strains in stress conditions, freeze-drying process and gastrointestinal tolerance were performed in this study. The experiments were performed as specified below, with the activities of key enzymes used as the evaluation standard.

### Freeze-Drying Process

Bacteria were incubated in the CK and G1 media at 37°C for 8 h. The cells were collected by centrifugation (4°C, 6000 × *g*, 5 min) and mixed with lyophilized protective medium (sterile 12% skim milk). After freezing at -20°C for 10 h, samples were freeze-dried at -55°C for 24 h. The freeze-drying products were then immediately suspended in sterile saline, and the activities of key glucose metabolism enzymes determined.

### Simulated Gastrointestinal Conditions

After incubation in the MRS medium for 8 h, the cells were collected by centrifugation (4°C, 6000 × *g*, 5 min), washed twice with sterile saline, and suspended in sterile saline at 10^10^ CFU/mL. The suspensions were used in intestinal stress and gastric stress experiments (10% inoculum). The simulated gastrointestinal conditions were created as described by Zhou et al. (2015) and Succi et al. (2017), with some modifications. To simulate the gastric juice, pepsin was added (Sigma-Aldrich, MO, United States) to the CK and G1 media for a final concentration of 10 U/mL; the pH was adjusted to 2.0 using 1 mol/L HCl. After inoculation with bacterial suspensions, the mixtures were incubated at 37°C. Aliquots were withdrawn after 0, 1.5, and 3 h, and viable bacterial counts were determined on MRS agar plates. To simulate the intestinal juice, bovine bile salts (3%; Sigma-Aldrich) and trypsin (5 U/mL; Sigma-Aldrich) were added to the CK and G1 media, and the pH adjusted to 7.5 using 1 mol/L NaOH. After inoculation with bacterial suspensions, the mixtures were incubated at 37°C. Aliquots were withdrawn after 0, 1.5, and 3 h, and viable bacterial counts were determined on MRS agar plates.

### Statistics

All experiments and analyses were performed in triplicate, and the data are presented as the mean values. Microsoft Office Excel 2010 and SPSS (17.0) software were used for data analysis. One-way ANOVA and independent-sample *t*-test were used for statistical analysis. The significance threshold was set at *P* < 0.05.

## Results

### Monosaccharide Composition of LBP

**FIGURE 1:**
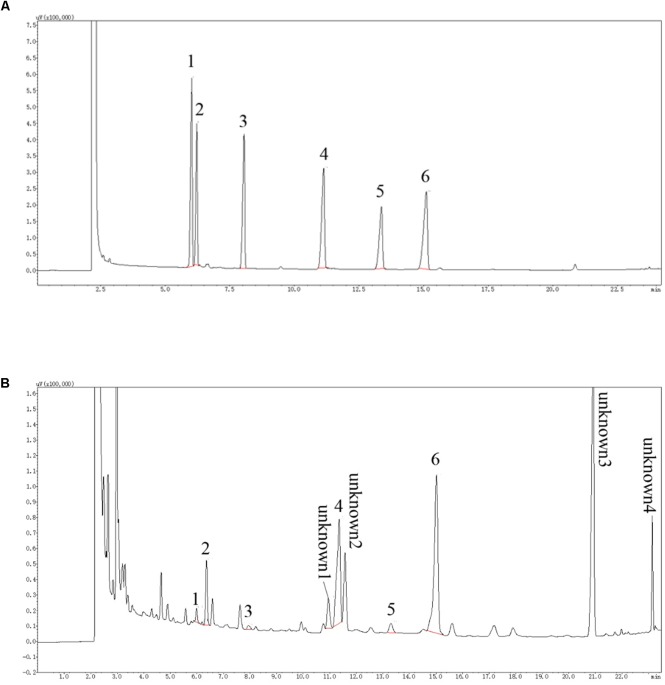
GC chromatogram of of monosaccharides. **(A)** represents GC chromatogram of standard monosaccharides, according to the peak order: 1 Arabinose, 2 Rhamnose, 3 Xylose, 4 Mannose, 5 Galactose 6 Glucose; **(B)** is the GC chromatogram of LBP sample monosaccharides. The monosaccharide corresponding to the standard is labeled with the same number. In addition, at least four kinds of unknown components in LBP with higher concentration were detected.

As determined by the GC analysis (**Figure 1**), LBP was composed of arabinose, rhamnose, xylose, mannose, galactose, and glucose, at molar ratios of 0.18:0.81:0.07:2.17:0.23:6.52. At least four unknown components with higher content were detected. The top three known monosaccharides were glucose (29.38%), mannose (9.88%), and rhamnose (3.34%).

### Proliferative Effect of LBP on LAB Strains

The growth profiles of *Bifidobacterium* strains and *Lactobacillus* strains in LBP-supplemented media are shown in **Table 1**. LBP improved the growth of all tested *Bifidobacterium* strains except for strain Bi-04. The viable counts of Bb-02, Bi-26, A6, and BY-02 strains in the G1 medium exceeded those on the CK medium after 4 h of growth. The growth rates of different *Bifidobacterium* strains in the G2 medium were different. The viable counts of *B. longum* subsp. *longum* A6 in the G2 medium were higher than those in the CK medium but lower than those in the G1 medium. Among the strains, the growth of *B. longum* subsp. *infantis* Bi-26 in the CK medium continuously increased over the 0–16 h period. However, cell numbers in the G1 and G2 media reached a maximum after 8 h, which indicated that LBP shortened the logarithmic growth phase and stable phase of growth of strain Bi-26. The data (**Table 1**) indicated that the G1 medium supported bacterial growth better than the G2 and CK media. Strain Bi-26 was chosen for further study, and henceforth cultured in the G1 medium.

**Table 1:** Growth of strains in different media.

Strain	Group	Colony forming units (log cfu/mL)				
		0 h	4 h	8 h	12 h	16 h
Bb-02	CK	7.07 ± 0.02	7.13 ± 0.01	8.89 ± 0.02	10.47 ± 0.06	10.05 ± 0.04
	G1	7.10 ± 0.03	8.43 ± 0.16*	8.96 ± 0.05	10.60 ± 0.09	10.45 ± 0.07*
	G2	7.05 ± 0.02	7.87 ± 0.17*	8.79 ± 0.11	10.54 ± 0.04	10.44 ± 0.13*
Bi-04	CK	7.79 ± 0.03	9.45 ± 0.11	10.03 ± 0.03	11.15 ± 0.13	11.17 ± 0.03
	G1	7.85 ± 0.06	9.41 ± 0.03	9.95 ± 0.04#	10.87 ± 0.11	11.21 ± 0.03
	G2	7.73 ± 0.10	9.46 ± 0.04	9.73 ± 0.01*	10.85 ± 0.09	11.13 ± 0.03
Bi-26	CK	7.43 ± 0.06	9.35 ± 0.08	10.11 ± 0.01	10.75 ± 0.07	10.95 ± 0.02
	G1	7.35 ± 0.07	9.66 ± 0.02*#	10.35 ± 0.04*#	11.17 ± 0.04*#	11.18 ± 0.02*#
	G2	7.51 ± 0.09	9.31 ± 0.02	10.08 ± 0.01	10.98 ± 0.06*	10.98 ± 0.01
A6	CK	7.62 ± 0.10	8.52 ± 0.22	8.94 ± 0.23	11.71 ± 0.03	10.54 ± 0.02
	G1	7.56 ± 0.06	9.25 ± 0.09*#	10.11 ± 0.03*#	11.81 ± 0.05	11.03 ± 0.07*
	G2	7.60 ± 0.04	8.63 ± 0.03	9.80 ± 0.06*	11.76 ± 0.06	10.74 ± 0.08
BY-02	CK	7.41 ± 0.07	9.27 ± 0.05	10.03 ± 0.10	10.63 ± 0.03	10.54 ± 0.03
	G1	7.35 ± 0.04	9.27 ± 0.16	10.12 ± 0.02	10.72 ± 0.05#	10.76 ± 0.05*
	G2	7.33 ± 0.03	9.09 ± 0.09	10.03 ± 0.00	10.18 ± 0.06*	10.51 ± 0.09
NCFM	CK	7.83 ± 0.05	9.35 ± 0.05	10.40 ± 0.01	11.05 ± 0.01	11.21 ± 0.01
	G1	7.75 ± 0.08	9.35 ± 0.10	10.89 ± 0.01*#	11.25 ± 0.01*#	11.26 ± 0.05
	G2	7.78 ± 0.04	9.34 ± 0.02	10.61 ± 0.04*	11.07 ± 0.02	10.92 ± 0.04
LP39	CK	7.91 ± 0.08	9.16 ± 0.12	10.80 ± 0.01	11.30 ± 0.02	11.28 ± 0.02
	G1	7.85 ± 0.03	9.22 ± 0.04	10.97 ± 0.01*#	11.52 ± 0.03*#	11.64 ± 0.02*#
	G2	7.86 ± 0.05	8.45 ± 0.15	10.24 ± 0.04*	11.31 ± 0.02	10.85 ± 0.03*

*All results were repeated three times and expressed as mean ± standard deviation of mean. ^∗^*P* < 0.05 G1 and G2 compared with CK, ^#^*P* < 0.05 G1 compared with G2*.

The optimal medium for the proliferation of *Lactobacillus* strains was also the G1 medium. As shown in **Table 1**, the viable counts of strain NCFM in the G1 medium reached 10.89 (log CFU/mL), which was significantly higher than those in the CK medium (*P* < 0.05). Similar data were observed for the LP39 strain. However, LBP exerted the most pronounced proliferative effect on strain NCFM, which was consequently used in subsequent experiments.

### Effect of LBP on the Activities of Key Glucose Metabolism Enzymes

**FIGURE 2:**
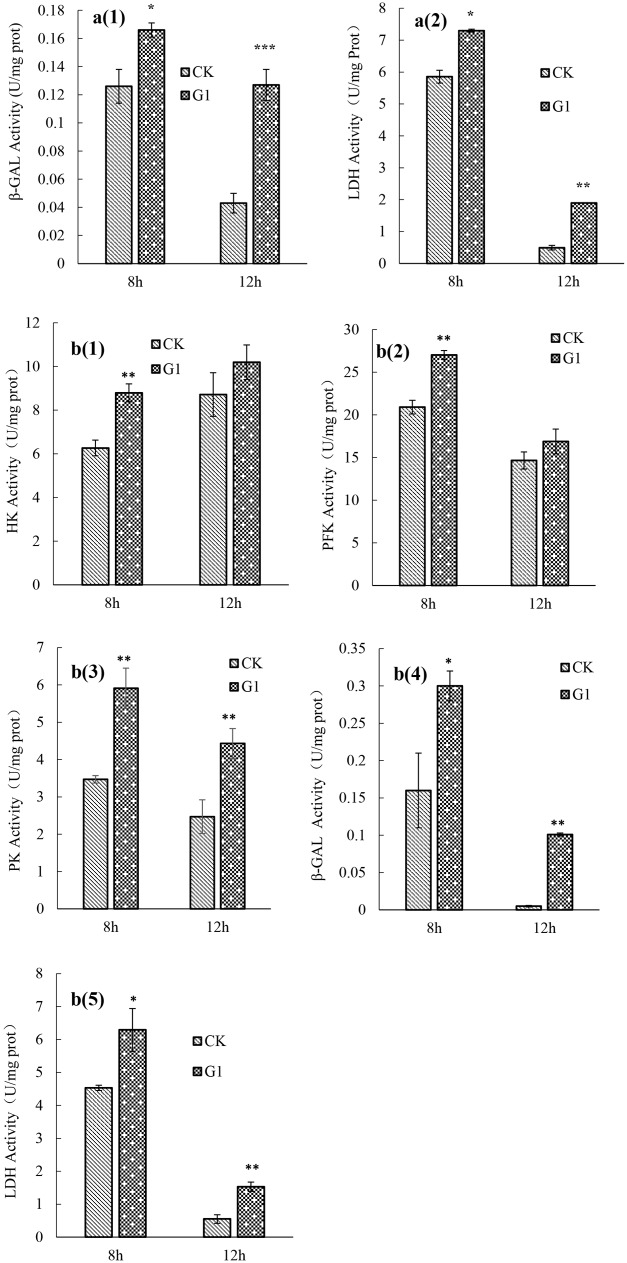
key enzymes activity of strain Bi-26 and NCFM in control and LBP-supplemented MRS broth incubated at 37°C for 8 and 12 h. **(a1)** was β-GAL activity of Bi-26, **(a2)** was LDH activity of Bi-26. **(b1)** represent HK activity of NCFM, **(b2)** was PFK activity of NCFM, **(b3)** was PK activity of NCFM, **(b4)** was β-GAL activity of NCFM, **(b5)** represent LDH activity of NCFM. Error bars represent standard deviation (*n* ≥ 3). ^∗^Means G1 significantly different from CK *P* < 0.05; ^∗∗^Means G1 Significantly different from CK *P* < 0.01; ^∗∗∗^Means G1 significantly different from CK *P* < 0.001.

The activities of key glucose metabolism enzymes were determined for *B. longum* subsp. *infantis* Bi-26 and *L. acidophilus* NCFM (**Figure 2**). The activities of key enzymes in strain Bi-26 and *L. acidophilus* NCFM were enhanced in the G1 medium. For strain Bi-26, the activities of β-GAL and LDH were higher after 8 h than after 12 h in this medium; for *L. acidophilus* NCFM, the activity of HK was higher at 12 h, but the activity of PFK, PK, β-GAL, and LDH was higher at 8 h.

### Transcriptome Analysis

#### Gene Ontology Annotation

**FIGURE 3:**
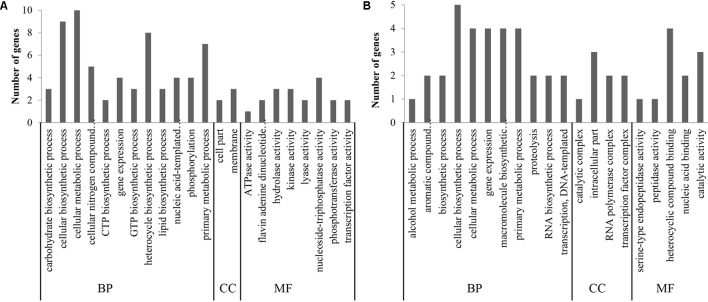
The most enriched GO terms. **(A)** represents the result of strain NCFM. **(B)** was the data of strain Bi-26. The unigenes were classified into three main categories [biological process (BP), cellular component (CC) and molecular function (MF)].

To classify the predicted functions of unigenes, the gene ontology approach was employed. The most enriched GO terms are shown in **Figure 3**. For strain NCFM (**Figure 3A**), the most enriched GO terms were the cellular metabolic process (GO:0044237), cellular biosynthetic process (GO:0044249), heterocycle biosynthetic process (GO:0018130), primary metabolic process (GO:0044238), cellular nitrogen compound metabolic process (GO:0034641), gene expression (GO:0010467), nucleic acid-templated transcription (GO:0097659), nucleoside-triphosphatase activity (GO:0017111), and phosphorylation (GO:0016310). For *B. longum* subsp. *infantis* Bi-26 (**Figure 3B**), the most enriched GO terms were the cellular biosynthetic process (GO:0044249), cellular metabolic process (GO:0044237), gene expression (GO:0010467), macromolecule biosynthetic process (GO:0009059), primary metabolic process (GO:0044238), heterocyclic compound binding (GO:1901363), intracellular part (GO:0044424), catalytic activity (GO:0003824), aromatic compound biosynthetic process (GO:0019438), proteolysis (GO:0006508), and transcription DNA-templated (GO:0006351).

### Metabolic Pathway Analysis

As shown in **Table 2**, four pathways were overexpressed and two pathways were suppressed when strain NCFM was grown in the presence of LBP. The overexpressed pathways were pyruvate metabolism, carbon metabolism, phosphotransferase system (PTS), and glycolysis/gluconeogenesis. The pentose phosphate pathway and biosynthesis of amino acids were suppressed in this strain. In *B. longum* subsp. *infantis* Bi-26, the overexpressed genes were involved in the pathways of pyruvate metabolism, metabolic pathways, carbon metabolism, and biosynthesis of secondary metabolites. The suppressed pathways in this strain were involved in thiamine metabolism and microbial metabolism in diverse environments.

**Table 2:** Pathway analysis of genes.

*L. acidophilus* NCFM		*B. longum* subsp. *infantis* Bi-26	
Over-expressed	Under-expressed	Over-expressed	Under-expressed
Pyruvate metabolism	Pentose phosphate pathway	Pyruvate metabolism	Thiamine metabolism
Carbon metabolism	Biosynthesis of amino acids	Metabolic pathways	Microbial metabolism in diverse environments
Phosphotransferase system (PTS)		Carbon metabolism	
Glycolysis / Gluconeogenesis		Biosynthesis of secondary metabolites	

### Differential Expression Analysis

As shown in **Table 3**, expression of seven genes was elevated in strain NCFM in the presence of LBP in the growth medium. These seven genes encoded phosphoenolpyruvate (PEP) synthase (*pps*), pyruvate formate-lyase-activating enzyme (*pflA*), formate C-acetyltransferase (*pflB*), nucleoside-diphosphate kinase (*ndk*), FAD-dependent glycerol-3-phosphate dehydrogenase (*glpD*), glycerol uptake facilitator protein (*glpF3*), and glycerol uptake facilitator protein (*glpF4*). The expression of four genes was reduced under these conditions. They were genes encoding small heat shock protein (*hsp1*), translation initiation factor IF-3 (*infC*), transketolase (*tkt2*), and cation (cobalt-zinc-cadmium) efflux protein (*czcD2*).

**Table 3:** Differential expression analysis.

Strain	Over-expressed	log2. Fold change	Under-expressed	log2. Fold change
NCFM	Phosphoenolpyruvate synthase (pps)	5.19	Small heat shock protein (hsp1)	2.53
	Pyruvate formate-lyase-activating enzyme (pflA)	3.57	Translation initiation factor IF-3 (infC)	2.12
	Formate C-acetyltransferase (pflB)	3.91	Transketolase (tkt2)	3.09
	Nucleoside-diphosphate kinase (ndk)	2.31	Cation (cobalt-zinc-cadmium) efflux protein (czcD2)	2.87
	FAD-dependent glycerol-3-phosphate dehydrogenase (glpD)	3.14		
	Glycerol uptake facilitator protein (glpF3)	2.65		
	Glycerol uptake facilitator protein (glpF4)	2.62		
Bi-26	Oligopeptide ABC transporter (oppA)	2.56	Transcription regulator of CopAB ATPases (copR)	3.25
	Dihydroxyacetone phosphotransferase (dak1A)	3.33	Thiamin pyrophosphokinase (tpk)	2.72
	Copper transporting ATPase (copA)	14.88	Xylulose-5-P phosphoketolase (xpkA)	4.19
	Copper transporting ATPase (copB)	12.79	Ribulose-phosphate 3-epimerase (rpe)	2.75
	Extracellular transglycosylase	2.32		

In strain Bi-26, five genes were overexpressed in the presence of LBP (**Table 3**). They encoded oligopeptide ABC transporter (*oppA*), dihydroxyacetone phosphotransferase (*dak1A*), copper transporting ATPase (*copA*), copper transporting ATPase (*copB*), and extracellular transglycosylase. The genes whose expression was reduced in this strain in the presence of LBP encoded transcription regulator of CopAB ATPases (*copR*), thiamin pyrophosphokinase (*tpk*), xylulose-5-P phosphoketolase (*xpkA*), and ribulose-phosphate 3-epimerase (*rpe*).

### The Protective Effect of LBP on LAB Strains Exposed to Stress Conditions

#### The Effect of LBP on Cell Survival After Freeze-Drying

The activities of key enzymes of *B. longum* subsp. *infantis* Bi-26 and *L. acidophilus* NCFM after freeze-drying showed the same tendency in the presence of LBP (**Table 4**). Compared with cells grown in the CK medium (data not shown), the activities of key enzymes were significantly increased (*P* < 0.05) after growth in the G1 medium. This indicated that LBP improved the tolerance of strains Bi-26 and NCFM to freeze-drying by protecting the activity of key cellular enzymes.

**Table 4:** The activities of key enzymes of strain Bi-26 and NCFM after freeze drying.

Group	Bi-26 (U/mg prot)				NCFM (U/mg prot)
	
	β-GAL	LDH	HK	PFK	PK	β-GAL	LDH
**CK**	0.184 ± 0.006^a^	5.323 ± 0.080^c^	7.260 ± 0.082^e^	12.661 ± 0.079^g^	4.803 ± 0.008^j^	0.128 ± 0.011^m^	2.330 ± 0.075^p^
**G1**	0.200 ± 0.008^b^	6.828 ± 0.119^d^	9.807 ± 0.065^f^	14.322 ± 0.113^h^	5.451 ± 0.012^k^	0.185 ± 0.016^n^	4.886 ± 0.094^q^

*All results were repeated three times and expressed as mean ± standard deviation of mean. Means in the same column with different superscripted letters are significantly different (*P* < 0.05)*.

#### The Effect of LBP on Cell Gastric and Intestinal Tolerance

**FIGURE 4:**
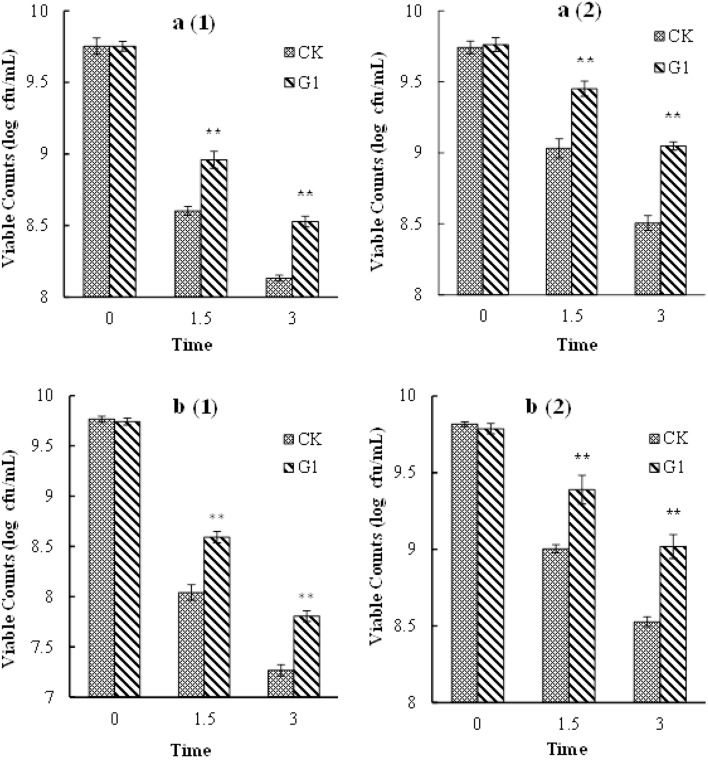
Gastric and intestinal fluids tolerance of Bi-26 and NCFM. **(a1)** shows viable counts in gastric fluid of Bi-26, **(a2)** represents viable counts in intestinal fluid of Bi-26. **(b1)** was viable counts in gastric fluid of NCFM, **(b2)** was viable counts in intestinal fluid of NCFM. Error bars represent standard deviation (*n* ≥ 3). ^∗^Means G1 significantly different from CK at same time *P* < 0.05;^∗∗^Means G1 Significantly different from CK at the same time *P* < 0.01.

The growth profiles of *B. longum* subsp. *infantis* Bi-26 and *L. acidophilus* NCFM in a simulated gastrointestinal environment are shown in **Figure 4**. The number of viable cells of strain Bi-26 and *L. acidophilus* NCFM was obviously reduced in the gastric fluid and intestinal fluid media. Bacterial tolerance of the gastric fluid medium was lower than that of the intestinal fluid medium. Compared with cells grown in the CK medium, the tolerance of the gastrointestinal environment of *B. longum* subsp. *infantis* Bi-26 and *L. acidophilus* NCFM cells was improved by the presence of LBP; the number of viable cells was significantly higher than in the CK medium cells (*P* < 0.05) after 1.5 h and 3 h of stress exposure.

## Discussion

In the current study, the effect of LBP on the proliferation and survival of LAB was examined. The presented data suggested that the LAB strains differ with respect to their ability to use LBP. This was because the strains produce different types of glycosidases, whose activities were also different. In the current study, LBP promoted the proliferation of all tested LAB strains except strain Bi-04. The growth of strain Bi-04 was inhibited by LBP, which may have been associated with a high osmotic pressure exerted by LBP, leading to bacterial dehydration and growth inhibition (Wan et al., 2007). *B. bifidum* Bb-02, *B. longum* subsp. *infantis* Bi-26, *B. longum* A6, *B. animalis* BY-02, *L. acidophilus* NCFM, and *L. plantarum* LP39 were able to effectively utilize LBP. In summary, LBP can be used by various LAB because it is composed of different monosaccharides; consequently, it may be used as a prebiotic by many LAB.

Generally, LAB or strains of the species *L. acidophilus* produce lactic acid by EMP pathway. As is generally known, β-GAL, HK, PFK, PK, and LDH are the most important enzymes of carbohydrate metabolism (Cheng et al., 2014). *Bifidobacterium* strains produce lactic acid and acetic acid, etc., by a special HMP pathway. β-GAL and LDH play an important role in metabolic processes. Some other *Bifidobacterium*-specific enzymes of sugar metabolism, such as α- and β-glucosidase, mannosidase, and xylosidase were also in strain Bi-26. In the current study, the activity of key glucose metabolism enzymes was enhanced by LBP. Carbohydrate metabolism significantly affects the growth of bacterial cell and the synthesis of secondary metabolites. The above-mentioned data indicated that LBP promotes the proliferation of *B. longum* subsp. *infantis* Bi-26 and *L. acidophilus* NCFM.

In strain NCFM, genes overexpressed in the presence of LBP encoded several proteins involved in carbon metabolism (Pps, Pfla, PflB, GlpD, GlpF3, and GlpF4) and Ndk. Pps plays an essential role in glycolysis in the modified Embden–Meyerhof–Parnas pathway, converting pyruvate to PEP. PEP is a precursor in several biosynthetic processes (Farmer and Liao, 2001). PflA and PflB belong to the radical *S*-adenosylmethionine family of enzymes; they play important roles in one-carbon unit transfer (Crain and Broderick, 2014). GlpD, GlpF3, and GlpF4 are involved in glycerol metabolism. GlpD catalyzes the conversion of glycerol-3-phosphate to dihydroxyacetone phosphate. It is involved in the utilization of glycerol coupled to respiration. GlpF3 and GlpF4 are membrane proteins of the aquaporin family. Ndk is required for the synthesis of nucleoside triphosphates (NTP). It provides NTPs for nucleic acid synthesis, CTP for lipid synthesis, UTP for polysaccharide synthesis, and GTP for protein elongation and signal transduction. The data indicated that carbohydrate and energy metabolism were induced in the NCFM cells by LBP. This demonstrated that LBP promoted the proliferation of that strain. On the other hand, the expression of *hsp1*, *infC*, *tkt2*, and *czcD2* genes was reduced in the presence of LBP (**Table 3**). Hsp1 is involved in the response to stress (e.g., temperature, hypertonicity, and amino acid deprivation). Its reduced gene expression indicated that LBP provided a uniform environment for bacterial cells. In other words, LBP enhanced the adaptability of cells to their microenvironment. Transketolase, encoded by *tkt2*, catalyzes the reversible transfer of a two-carbon ketol unit from xylulose 5-phosphate to an aldose receptor. It plays an important role in the pentose phosphate pathway, which is a major source of the reducing power and metabolic intermediates for biosynthetic processes. Hence, reduced expression of the transketolase gene indicated that the cells absorbed various nutrients from the medium in the presence of LBP. Further, CzcD2 is thought to be an efflux pump that removes cobalt, zinc, and cadmium ions from the cell. These results suggested that LBP increased the anabolism and promoted cell proliferation.

In *B. longum* subsp. *infantis* Bi-26, the following genes were overexpressed in the presence of LBP: *opp*, *dak1A*, *copA*, *copB*, and extracellular transglycosylase (**Table 3**). Transmembrane transport of oligopeptides requires the transport of a solute across a lipid bilayer. Opp is an ATP-dependent oligopeptide transporter and a member of the ATP-binding cassette (ABC) superfamily of transporters (Turroni et al., 2010). Since the gene *oppA* was overexpressed in Bi-26 cells in the presence of LBP (**Table 3**), this indicated that LBP promoted protein import. Dak1A converts dihydroxyacetone to the glycolytic intermediate dihydroxyacetone phosphate (Siebold et al., 2003). CopA is a protein with a copper-exporting ATPase activity, while CopB possesses a copper-importing ATPase activity. CopA and CopB can facilitate the transfer of a solute or solutes across the membrane. Extracellular transglycosylase plays an important role in the biosynthesis of peptidoglycan. Collectively, these data indicated that LBP enhanced the formation of the cell wall in strain Bi-26. Among the genes whose expression was reduced in Bi-26 cells in the presence of LBP were *copR*, *tpk*, and *xpkA*. CopR is a DNA-templated negative regulator of transcription. Phosphoketolases are key enzymes of the pentose phosphate pathway in heterofermentative and facultative homofermentative LAB. XpkA is a thiamine diphosphate (ThdP)-dependent enzyme found in *Bifidobacterium* sp. (Yin et al., 2005). The data therefore indicated that LBP stimulated the propagation of strain Bi-26 by enhancing the intracellular transport and synthesis.

Freeze-drying is one of the most effective methods for preserving microbial strain seeds. However, inevitably, cell viability is reduced during this process. As shown in the current study, LBP effectively improved the cell viability during freeze-drying. Carvalho et al. (2004) reported that the survival of LAB strains during and after vacuum freeze-drying depended on the sugars present in the growth and drying media. Specific sugars, other than glucose, might underpin the distinct cell survival behavior during freeze-drying. As demonstrated in the current study, LBP mainly consists of arabinose, rhamnose, xylose, mannose, galactose, and glucose. Specific sugars might be produced by LAB strains during growth in a medium containing LBP that might exert a protective effect on LAB strains during freeze-drying. The protective effect may be also associated with the spatial distribution of the hydroxyl groups. The massive hydroxyl groups replace water molecules and maintain the spatial structure of bacterial proteins by interacting with the polar groups of protein via hydrogen bonds. This prevents protein denaturation associated with dehydration during freeze-drying (Carvalho et al., 2003; Yue et al., 2016).

To confer a health benefit on the host, the LAB must be able to overcome the physical and chemical barriers of the gastrointestinal tract, especially acid, proteolytic enzyme, and bile stresses (Nami et al., 2017). Consequently, LAB should be resistant to the gastrointestinal environment. As demonstrated in the current study, LBP improved the tolerance of *B. longum* subsp. *infantis* Bi-26 and *L. acidophilus* NCFM to the gastrointestinal environment. In fact, numerous studies indicate that some prebiotics are able to exert a positive effect on the viability of LAB (Oliveira et al., 2009; Rattanaprasert et al., 2014; Succi et al., 2017). However, it remains unclear how prebiotics are able to affect the growth and resistance of LAB exposed to stress environments. From the previous reports (Even et al., 2002; Blaiotta et al., 2013), the possible mechanism was that the activities of key glucose metabolism enzymes were improved by LBP, it was helpful for fast recovery of bacteria. The hydrophobic effect of bile salts was also reduced by LBP. These effects were beneficial for LAB, and enhanced their tolerance of the gastric fluid and intestinal fluid media.

## Conclusion

LBP is a special carbon source, which contains arabinose, rhamnose, xylose, mannose, galactose, and glucose. It promotes the proliferation of some *Bifidobacterium* and *Lactobacillus* strains, possibly by enhancing the carbon and energy metabolism. It also exerts a protective effect on LAB strains exposed to stress conditions. Hence, LBP can be used as an industrial prebiotic candidate.

## Author Contributions

FZ conducted the experiments and wrote the manuscript. XJ and TW conducted the experiments. BZ provided some suggestions. HZ provided some ideas and checked the manuscript.

## Conflict of Interest Statement

The authors declare that the research was conducted in the absence of any commercial or financial relationships that could be construed as a potential conflict of interest. The reviewer PM and handling Editor declared their shared affiliation.

## Footnotes

1 https://www.blast2go.com
